# Clinical Management of Metabolic Syndrome Among the Population Attending Geriatric Outpatient Clinics in Qatar

**DOI:** 10.7759/cureus.67826

**Published:** 2024-08-26

**Authors:** Susan Mohieldeen Osman, Brijesh Sathian, Biju Bhaskaran, Marwan Ramadan, Haroon Saleh, Asma Abbas, Hanadi Al Hamad

**Affiliations:** 1 Geriatrics and Long-Term Care Department, Rumailah Hospital, Hamad Medical Corporation, Doha, QAT

**Keywords:** older adults, obesity, hypertension, diabetes mellitus, metabolic syndrome

## Abstract

Introduction: Research on the clinical management of metabolic syndrome (MetS) among older individuals in Qatar is limited. This study aimed to determine the clinical management of MetS and associated risk factors.

Methods: A retrospective study was conducted to examine the risk factors for MetS (hypertension, diabetes mellitus, obesity, and hyperlipidemia) among patients aged ≥ 60 years who visited geriatric outpatient clinics in Rumailah Hospital, Doha, Qatar between November 1, 2016, and November 1, 2018.

Results: The mean age of the patients was 70.1 years, and 50% were male. Of the study population, 97% had MetS with a 95% confidence interval (95.3-98.7). In addition, 45.5% of the patients were obese, 97.75% had diabetes mellitus, and 98.25% had hypertension. The most commonly prescribed medications for treatment included amlodipine for hypertension, metformin for diabetes mellitus, rosuvastatin for lipid reduction, and bisoprolol for cardiovascular management.

Conclusion: This study found that MetS is common among the population attending geriatric outpatient clinics in Qatar. The majority of these individuals had high rates of diabetes, hypertension, and dyslipidemia. The early identification of at-risk patients through exercise programs may also delay or reverse the risks associated with MetS. More research, especially prospective and population-based studies, is required to improve preventative efforts and optimize treatment options for metabolic syndrome in older persons in Qatar.

## Introduction

Globally, the aging population is rapidly changing, and the percentage of people over 60 years of age is expected to increase from 12% to 22% between 2015 and 2050. [1]. Risk factors for chronic illness increase with age. As a result, the prevalence of metabolic syndrome (MetS) syndrome will increase worldwide, increasing its importance in the health system. [1,2].

MetS is a cluster of physiological and metabolic risk factors associated with an increased risk of serious health conditions, such as cardiovascular disease (CVD), type 2 diabetes, and stroke. It has been recognized as a proinflammatory and prothrombotic state, and these characteristics were identified by Reaven three decades ago [3]. Although originally known as syndrome X, it was later referred to as "plurimetabolic syndrome" or "Reaven’s syndrome." Since the observation that insulin resistance underlies syndrome X, it has been termed insulin resistance syndrome [4]. In 1967, Avogaro et al. described it as "metabolic syndrome" [5].

The presence of three or more of the five risk factors, namely, raised levels of fasting blood glucose, blood pressure (BP), triglycerides, and low high-density lipoprotein, as well as obesity (particularly central adiposity) indicate a diagnosis of MetS. Obesity is the most common risk factor for MetS [5]. The definition of the syndrome is currently being debated [5]. The most recent and commonly used definitions were developed by the International Diabetes Federation (IDF) and the American Heart Association/National Heart, Lung, and Blood Institute AHA/NHLBI [6-8].

Sliem et al. conducted a systematic review of MetS in the Middle East, which showed that its prevalence ranges from 15-60% among the Middle Eastern population [9]. There was a female predominance. Qatar National Stepwise Survey reported that the prevalence of MetS increased with age from 9.3% in those aged <30 years to 70.2% in those aged 60-64 years [10].

MetS is strongly associated with a higher risk of suicide, especially in people with serious mental illnesses. A recent study showed that individuals with mental health disorders such as schizophrenia have a greater occurrence of MetS, leading to higher rates of heart-related deaths and shorter lifespans [11]. A study conducted within a community discovered that every element of MetS increased the likelihood of suicide by 16%, and high blood pressure stood out as a significant individual risk factor [12]. Moreover, research conducted on hospitalized patients found that 59.4% faced depression and 50% reported having suicidal thoughts, emphasizing the connection between physical health and emotional wellness [13].

There are no studies from Qatar in the older adult population with MetS, and this vulnerable population has more comorbidities. The objective of our study was to determine the clinical management of MetS in older adult patients attending medical outpatient clinics at Rumailah Hospital in Qatar. 

## Materials and methods

Study design and participants

This retrospective study aimed to identify the risk factors related toMetS among older adults aged 60 or above who attended geriatric outpatient clinics at Rumailah Hospital in Doha, Qatar, during the period from November 1, 2016, to November 1, 2018. A total of 400 participants were included in the study, based on an estimated prevalence of MetS among adults of 30% with a 5% absolute error and a 95% confidence interval (CI), requiring a sample size of 350. The study targeted a slightly larger sample to account for potential data loss or inaccuracies.

Data collection

The data used in the study were obtained from the Cerner electronic medical records system maintained by Hamad Medical Corporation. The collected data included patient demographics, such as age and sex, as well as cardiovascular disease (CVD) risk factors and the presence of various metabolic conditions. Specifically, data on antihypertensive medications, fasting blood glucose levels, triglycerides, high-density lipoprotein (HDL) cholesterol levels, and body mass index (BMI) were obtained.

Criteria for diagnosing MetS

The criteria for diagnosing MetS are based on the presence of three or more of the following five risk factors: (1) Raised fasting blood glucose : ≥100 mg/dL or ≥5.6 mmol/l, or a diagnosis of diabetes.; (2) Raised triglycerides: ≥150 mg/dL or ≥1.7 mmol/l, or drug treatment for hypertriglyceridemia; (3) reduced in HDL cholesterol: <40 mg/dL in men or <50 mg/dL in women.; (4) Obesity: BMI ≥30 kg/m²; (4) raised BP: systolic BP ≥130 mmHg or diastolic BP ≥85 mmHg, or the use of antihypertensive medication [9, 10].

The methodology described above provides a thorough approach to understanding the prevalence and management of MetS among older adults in a tertiary care setting in Qatar, offering valuable insights for healthcare planning and intervention strategies tailored to this population.

Data processing and statistical analysis

The obtained data was carefully inspected, coded, and recorded into a secure database on a password-protected computer at Rumailah Hospital.

A competent research team input and processed the data to ensure its quality and consistency. Anonymization procedures were applied to safeguard the participants' privacy and identities. Descriptive and inferential statistics were employed to summarize the data. Mean was calculated for continuous variables, while frequency and percentages were computed for categorical variables. Confidence intervals (CI) at 95% were applied to provide precision estimates. Additionally, the data was stratified by age groups (60-69, 70-79, and 80-90 years) to evaluate the variations in MetS prevalence and risk factors across different age brackets. The chi-square test results were utilized to identify any significant associations between the age group and the prevalence of MetS and its components. A p-value of less than 0.05 was considered statistically significant. R.3.5.2 software (R Foundation for Statistical Computing, Vienna, Austria) was used for all statistical analyses.

Ethical considerations

The study was reviewed and approved by the Institutional Review Board (IRB) of Hamad Medical Corporation (MRC-01-19-041). All procedures performed in the study involving human participants were in accordance with the ethical standards of the institutional research committee and the 1964 declaration of Helsinki. In addition, this study was funded by the Medical Research Center of Hamad Medical Corporation (MRC-01-19-041). To ensure anonymity, all participant data, including name and contact information, was deidentified. The dataset was only accessible to authorized members of the study team, and all data was stored securely to avoid unauthorized access. The institutional review board waived the necessity for informed consent.

## Results

Demographic and clinical characteristics

The study included 400 patients aged ≥ 60 years, with a mean age of 70.1 years. The demographic and clinical characteristics of the study population are summarized in Table 1. Of the total participants, 50.3% (n=201) were male and 49.7% (n=199) were female. The majority of patients were Qatari nationals, comprising 56.5% (n=226) of the study population. Medications prescribed to the study population are listed in Table 2. 

**Table 1:** Demographic and clinical characteristics The data was represented as a number (percentage)

Variables	n (%)
Gender	
Female	199 (49.75%)
Male	201 (50.25%)
Nationality	
Qatari	226 (56.50%)
Metabolic syndrome	388 (97%)
Risk factors	
Diabetic mellitus	391 (97.75%)
Obesity	182 (45.5%)
High triglycerides	371 (92.77%)
Low HDL	378 (94.74%)
Hypertension	393 (98.25%)

**Table 2:** Medications prescribed to the patient population in our study

Medications	n (%)
Diabetic medications	
Dapagliflozin	20 (5.87%)
Biphasic-insulin	14 (3.98%)
Aspart-insulin	52 (14.8%)
Degludec-insulin	2 (0.57%)
Dulaglutide	7 (1.99%)
Exenatide	2 (0.57%)
Glargine-insulin	95 (26.99%)
Glibenclamide	8 (2.27%)
Glimepiride	40 (11.36%)
Gliclazide	96 (27.27%)
Glipizide	10 (2.84%)
Isophane-insulin	5 (1.4%)
Liraglutide	13 (3.69%)
Lispro-insulin	20 (5.68%)
Metformin	274 (78%)
Mixtard-insulin	1 (0.28%)
Sitagliptin	138 (39.2%)
Repaglinide	3 (0.84%)
Pioglitazone	17 (4.75%)
Vildagliptin	34 (9.66%)
Anti-hypertensives	
Felodipine	12 (3.14%)
Fosinopril	6 (1.57%)
Hydralazine	11 (2.88%)
Hydrochlorothiazide	51 (13.35%)
Indapamide	48 (12.57%)
Irbesartan	36 (9.58%)
Captopril	1 (0.26%)
Amlodipine	117 (30.63%)
Arginine	11 (2.8%)
Enalapril	21 (5.50%)
Losartan	25 (6.54%)
Moxonidine	6 (1.57%)
Nifedipine	11 (2.88%)
Perindopril	65 (17.02%)
Valsartan	110 (28.8%)
Phentolamine	1 (0.25%)
Chlorthalidone	2 (0.52%)
Cilazapril	2 (0.52%)
Clonidine	1 (0.26%)
Cholesterol-lowering medication	
Atorvastatin	219 (61%)
Ezetimibe	2 (0.55%)
Fluvastatin	1 (1.94%)
Rosuvastatin	105 (29.09%)
Simvastatin	16 (4.4%)
Pravastatin	12 (3.2%)
Cardiac medication	
Bisoprolol	65 (16.75%)
Bumetanide	1 (0.26%)
Atenolol	50 (13.09%)
Carvedilol	18 (4.7%)
Diltiazem	7 (1.84%)
Furosemide	41 (10.73%)
Metoprolol	32 (8.38%)
Nebivolol	1 (0.25%)
Verapamil	3 (0.78%)
Triamterene	1 (0.25%)
Propranolol	7 (1.8%)
Supplement	
Omega 3	8 (2.22%)

Risk factors

Among the study population, 97.75% (95% CI: 95.8-99.1%) were diagnosed with diabetes mellitus, 45.5% (95% CI: 40.6-50.4%) were classified as obese, 92.77% (95% CI: 90.3-95.2%) had elevated triglyceride levels, 94.74% (95% CI: 92.5-96.9%) had low HDL cholesterol levels, and 98.25% (95% CI: 96.8-99.7%) had hypertension.

Age group comparison

Table 3 illustrates the age group-wise comparison of the risk factors for MetS. The percentage of obese patients was the highest in the 60-69 years age group at 53.37% (95% CI: 46.5-60.2%) and decreased with age, with 39.49% (95% CI: 32.0-47.3%) in the 70-79 years group and 34.00% (95% CI: 21.2-48.8%) in the 80-90 years group. The prevalence of diabetes was consistently high across age groups, with a slight decrease in the oldest group at 96% (95% CI: 86.3-99.5%). Low HDL cholesterol and hypertension were prevalent in over 94% of the study population across all age groups, with hypertension being the highest in the 80-90 age group at 98% (95% CI: 89.2-99.9%). High triglyceride levels were present in over 90% of patients in each age group. The results of the chi-square test indicated that there was a significant relationship between age groups and obesity (p = 0.008), but no significant associations were identified between age groups and the other components of MetS, including diabetes, low HDL cholesterol, hypertension, and high triglycerides.

**Table 3:** Comparison of MetS and its risk factors according to age group The data were represented as a number (percentage), and the association was represented as a p-value, where the p-value was considered statistically significant when it was less than 0.05.

Parameters	n	Metabolic syndrome; n (%)	Obesity; n (%)	Diabetes; n (%)	Low HDL; n (%)	Hypertension; n (%)	High triglycerides; n (%)
60-69 years	193	186 (96.37%)	103 (53.37%)	191 (98.96%)	182 (94.30%)	190 (98.45%)	178 (92.23%)
70-79 years	157	153 (97.45%)	62 (39.49%)	152 (96.82%)	148 (94.27%)	154 (98.09%)	148 (94.27%)
80-90 years	50	49 (98.00%)	17 (34.00%)	48 (96.00%)	48 (97.96%)	49 (98.00%)	45 (90.00%)
Total	400	388 (97.00%)	182 (45.50%)	391 (97.75%)	378 (94.74%)	393 (98.25%)	371 (92.75%)
P-values		0.762	0.008	0.271	0.559	0.959	0.555

## Discussion

Older adults with multiple comorbidities often have MetS and thus have an increased risk of cardiovascular disease. Health professionals depend on the prevalence and risk factors of MetS to plan and develop primary and secondary prevention strategies aimed at reducing serious adverse outcomes associated with MetS and its risk factors in older adults. Hence, it is extremely important that the estimated prevalence of MetS in older adults be reliable and based on information that is appropriate for the country. This study aimed to quantify the prevalence of MetS and its risk factors in the Qatar older adult population based on data from a subgroup of patients attending the geriatric outpatient clinic at the Rumailah Hospital in Qatar.

The outpatient-based prevalence of MetS in our study population was 97%. According to our study, regardless of age group, the prevalence of individual risk factors of MetS, namely hypertension, diabetes mellitus, hypertriglyceridemia, and low HDL, were all high, whereas obesity was low, especially in the age groups of 70-90. The prevalence of obesity was significantly higher in females (64%) than in males (27%). There was no significant difference in the age-group-wise prevalence of MetS between men and women in other studies. This might be due to the subgroup with very high-risk cardiovascular risk factors, and the subsequent increase in comorbidities might have led to a lack of physical activity, mobility limitation, and a sedentary lifestyle [14-16].

The prevalence of MetS often varies depending on the criteria and age. The worldwide prevalence of MetS in older adults varies from 30-70% [17, 18]. Aging is associated with an accumulation of comorbidities, including cardiovascular comorbidities, and a higher incidence of MetS. It is well known that MetS is associated with an increased risk of cardiovascular diseases and related mortality, both in the general population and in older adults. It is also well established that individual components of MetS are associated with increased mortality in the general population. However, obesity in older adults has been associated with a lower risk of mortality. Moreover, results from a large retrospective cohort study concluded that the individual components of MetS were better predictors of mortality [19].

The prevalence of MetS appears to be very high in our study compared to other studies, and this could be related to a selection bias [13]. This study used data from a cohort of older adults attending geriatric medicine outpatient clinics at our hospital, which might indicate that this subgroup could be associated with a higher incidence of comorbidities in general. However, we also note that epidemiological data from Qatar and other Gulf Cooperation Council countries show a higher incidence and prevalence of CVD risk factors, especially diabetes mellitus, compared to other parts of the world [9, 13]. Given this, it is likely that the older adult population in Qatar might have an increased prevalence of MetS, and the associated risk factors and findings from our study confirm a similar trend.

Our study also showed that the prevalence of obesity among the MetS risk factors was significantly lower than that of the other MetS components. According to data for Qatar from the Global Obesity Observatory, the prevalence of obesity in adults aged 45-64 years appears to be increasing over the last two decades. In 2003, the prevalence of obesity was 34.3% in males and 45.3% in females in the above age group; in 2012, it increased to 41.4% and 65%, respectively. However, it is noteworthy that the prevalence of obesity appeared to be much lower in the older adult population in our study than in the 45-64-year age group. This difference could be linked to multiple factors, including greater multimorbidity and associated frailty.

Identifying the prevalence of MetS and its associated components in older adults would be of crucial significance, as this would help manage individual risk factors more appropriately and reduce the associated adverse outcomes. Recent studies have suggested that an approach targeting individual components is more effective than addressing MetS as a whole.

It is necessary to conduct further population-based, translational, and randomized controlled trials to investigate the effective interventions to manage and prevent MetS. More studies are needed to understand the prevalence and effect of MetS in older adults and to detect biomarkers linking MetS to chronic diseases, sarcopenia, fatty liver, and cancer. In addition, innovative trials are required to identify if the effectiveness of interventions, including resistance exercise and a high-protein diet to improve muscle strength, will lead to a lower prevalence of MetS. We also aimed to understand the impact of preventive interventions on reducing the adverse events associated with MetS in older adults.

There is recent evidence accumulating from various prevention studies that address vascular risk factors, and positive modifications of lifestyle factors are associated with a significant reduction in various conditions, such as cardiovascular events and cognitive decline (FINGER trial) [20]. In addition, the study showed a high prevalence of MetS and its components in the older adult Qatari population, which is similar to data from other studies.

Limitations

This research has several limitations that require acknowledgment. First, the use of a retrospective design restricts the ability to determine causality between risk factors and outcomes since historical data may contain inaccuracies or incomplete records. Secondly, the study population was limited to patients attending geriatric outpatient clinics at Rumailah Hospital, which may introduce potential selection bias as this subgroup may not be representative of the broader older adult population in Qatar. Thirdly, the single-center design of the study restricts the generalizability of the findings to other healthcare settings or regions. The absence of a control group also impedes comparative analyses with non-attending populations or different age groups. Data extracted from electronic medical records may contain documentation errors or missing information, which could affect accuracy. Furthermore, the study's retrospective design only provides a snapshot of the prevalence and management of MetS, without accounting for longitudinal changes. Potential confounding variables, such as socioeconomic status and genetic predispositions, may not have been fully addressed. Another limitation was the age of the secondary data. Finally, the study's focus on clinical management excludes consideration of non-clinical factors that could impact the management and outcomes of MetS in older adults, thus limiting the comprehensiveness of the findings. 

Large sample sizes and extensive data collection on the clinical management of MetS and risk variables in the elderly give valuable insights, particularly through age-group-specific analysis. The major findings, such as the high prevalence of diabetes and hypertension, emphasize the population's health burden. Furthermore, focusing on the Qatari senior population adds significant regional importance.

## Conclusions

This study found that MetS is common among the population attending geriatric outpatient clinics in Qatar. The majority of these individuals had high rates of diabetes, hypertension, and dyslipidemia. Therefore, it is crucial to identify patients at risk as early as possible. More research, especially prospective and population-based studies, is required to improve preventative efforts and optimize treatment options for metabolic syndrome in older persons in Qatar.
